# The Effect of Prone Position on Right Ventricular Functions in CARDS: Is Survival Predictable when Evaluated Through Transesophageal Echocardiography?

**DOI:** 10.4274/TJAR.2025.241830

**Published:** 2025-03-21

**Authors:** Dicle Birtane, Zafer Çukurova, Sinan Aşar, Damla Özmen, Gökhan Sertcakacılar, Fatma Nihan Çağlar Turhan

**Affiliations:** 1 University of Health Sciences Türkiye, Bakırköy Dr. Sadi Konuk Training and Research Hospital, Clinic of Anaesthesiology and Reanimation, Intensive Care Unit, İstanbul, Türkiye; 2 University of Health Sciences Türkiye, Bakırköy Dr. Sadi Konuk Training and Research Hospital, Clinic of Anaesthesiology and Reanimation, İstanbul, Türkiye; 3 Outcomes Research Consortium, Houston, Texas, USA; 4 University of Health Sciences Türkiye, Bakırköy Dr. Sadi Konuk Training and Research Hospital, Clinic of Cardiology, İstanbul, Türkiye

**Keywords:** ARDS, lung compliance, prone position, respiratory mechanics, right ventricular, transesophageal echocardiography

## Abstract

**Objective:**

To evaluate the cardiopulmonary effect during prone position (PP) on right ventricular (RV) recovery in coronavirus disease-2019 related acute respiratory distress syndrome (C-ARDS) through transesophageal echocardiography (TEE).

**Methods:**

This prospective study included 30 moderate-to-severe C-ARDS patients who were treated with PP in the first 48 h of invasive mechanical ventilation support. It was evaluated with TEE three times: before PP (T_0_), the first hour of PP (T_1_), and the first hour of returning to the supine position (T_0_ + 24 h) (T_2_) after 23 hours of PP treatment. RV end-diastolic area/left ventricular (LV) end-diastolic area (RVEDA/LVEDA), tricuspid annular plane systolic excursion (TAPSE) and LV end-systolic eccentricity index were preferred RV evaluations as primary outcomes. Pulmonary effects of PP were evaluated as a secondary outcome, including PaO_2_/FiO_2_, driving pressure (dP), static compliance (Cstat), mechanical ventilation parameters, and their association with 28-day survival. Tissue DO_2_ was examined as a secondary outcome, and it was calculated using the measured cardiac output through TEE.

**Results:**

With the cardiopulmonary effect of PP, the decrease in RVEDA/LVEDA, the increase in TAPSE, PaO_2_/FiO_2_, and Cstat, and the decrease in dP were statistically significant (*P* < 0.05). The Cstat value associated with 28-day survival showed decreased mortality for each unit increase. The Cstat cut-off value, which was statistically significant for survival, was 37.

**Conclusion:**

PP can improve RV recovery and oxygenation, but it isn’t always accompanied by increased survival. An increase in the Cstat may improve survival without the development of RV dysfunction while maintaining heart-lung interaction.

**Main Points**

• Prone position (PP) can improve right ventricular (RV) recovery and oxygenation but it isn’t always accompanied by increased cardiac output and DO_2_.

• The left ventricular (LV) curative effect of PP can be observed when LV function worsens secondary to RV dysfunction.

• The importance of the compatible lung can be explained both by its protective effect on the lung, preventing pressure and volume damage, and by its protective effect on the heart through the heart-lung interaction.

## Introduction

Non-coronavirus disease-19 (non-COVID-19) associated acute respiratory distress syndrome (ARDS) patients in the prone position (PP) showed improved right ventricular (RV) function by reducing RV pressure with effects on ventilation and gas exchange.^1^ In COVID-19 related ARDS (C-ARDS), especially in the severe form, increased shunt rate, impaired ventilation/perfusion ratio (V/Q), hypoxic pulmonary vasoconstriction inhibition, and increased immune microthrombosis may have similar effects on the RV.^2^ The cardiopulmonary pathophysiology and outcomes of C-ARDS vary, and this variability requires monitoring to follow the diagnosis and treatment process. This study aimed to increase the treatment success of the PP in C-ARDS and to provide a prognostic factor for survival by analyzing and monitoring heart-lung interactions. Therefore, we used transesophageal echocardiography (TEE) to evaluate the cardiopulmonary effects of PP.

The primary outcome of the study was that in patients with moderate/severe C-ARDS, improvement was observed in the RV with PP, i.e., there was a decrease in the RV end diastolic area/left ventricular (LV) end diastolic area (RVEDA/LVEDA) values at PP+1 h (T_1_) and PP+24 h (T_2_) compared to the pre-PP (T_0_) values, and this decrease can be used as a prognostic factor for survival. The secondary outcomes of this study were to analyze the cardiopulmonary effects of PP; to determine the changes in cardiac output (CO), LV end systolic eccentricity index (LVESEI), tricuspid annular plane systolic excursion (TAPSE), PaO_2_/FiO_2_, static compliance (Cstat), and dynamic compliance (Cdyn); and to investigate the relationship between these variables and prognostic factors.

## Methods

### Study Design and Population

**Figure 1:**
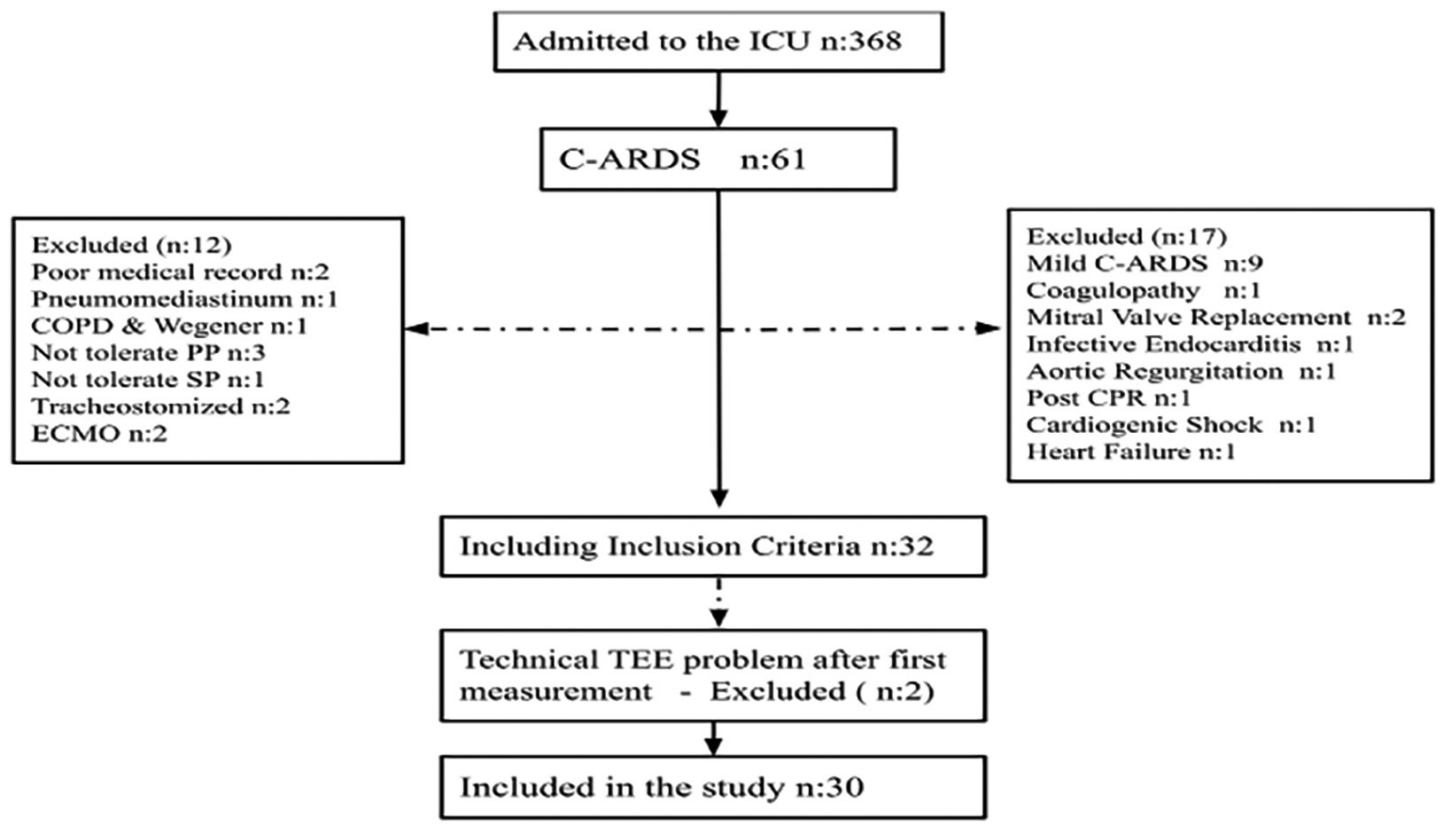
Flow diagram for the study ICU, intensive care unit; C-ARDS, coronavirus disease-2019 related ARDS; CPR, cardiopulmonary resuscitation; COPD, chronic obstructive pulmonary disease; ECMO, extracorporeal membrane oxygenation; TEE, transesophageal echocardiography

This study had a prospective, cross-sectional, single-center design. After obtaining ethical approval for the study, moderate/severe C-ARDS patients admitted to the University of Health Sciences Türkiye, Bakırköy Dr. Sadi Konuk Training and Research Hospital, Anaesthesiology and Reanimation Clinic Intensive Care Unit, who received invasive mechanical ventilation support and applied PP in the first 48 h, between February and May 2022, were included. The number of patients in the study was determined to be 30 based on the power analysis. The inclusion criteria were: i) age greater than 18 years; ii) patients diagnosed with polymerase chain reaction/computed tomography (PCR/CT) results, with moderate/severe severity class according to the Berlin ARDS classification, with prone positioning applied within the first 48 hours after orotracheal intubation; and iii) obtaining an informed consent form. A total of 30 patients were included. Exclusion criteria were as follows: relative, absolute contraindications for PP, and TEE, and a diagnosis of pulmonary embolism. It is shown in the flow chart (Figure 1).

### Ethical Consideration

This study was approved by the University of Health Sciences Türkiye, Bakırköy Dr. Sadi Konuk Training and Research Hospital, Clinical Research Local Ethics Committee with the decision number 2022-03-03, dated 07.02.2022, following the approval of the Ministry of Health Clinical Research Board form’-2022-01-30T12_22_28’ and was registered at clinical trials.gov (no: NCT06456606, protocol ID: 2022/40) by the principal investigator and was conducted in accordance with the Declaration of Helsinki, 2013.

### Data Collection

**Figure 2:**
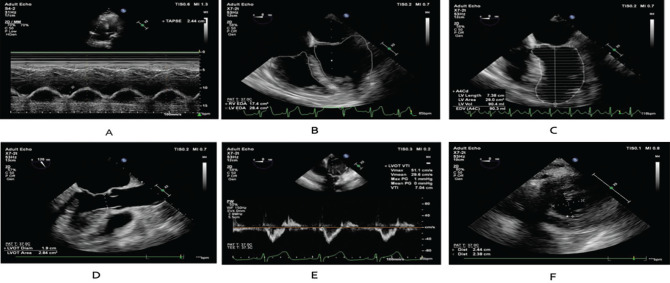
Transthorasic and transesophageal measurements A) TAPSE, B) LVEDA/RVEDA, C) LVEDV, D) LVOT diameter, E) LVOT-VTI, F) LVESEI TAPSE, tricuspid annular plane systolic excursion; LVEDA, left ventricular end diastolic area; RVEDA, right ventricular end diastolic area; LVEDV, left ventricular end diastolic volume; LVOT, left ventricular outflow tract; LVOT-VTI, left ventricular outflow tract velocity time integral; LVESEI, left ventricular end systolic eccentricity index

The patients were evaluated with TEE (x7-2t transducer with Philips Affiniti 30 System-Philips Healthcare, andover, MA, USA) in the supine position at three different times: before PP (T_0_), at the first hour of PP (T_1_, T_0_+1 h), and at the first hour of returning to supine after 23 h of PP (T_2_, T_0_+24 h). Each measurement was repeated three times by the same doctor, and the average values were recorded. TEE was performed by D.B. who has 5 years of experience using echocardiography in the intensive care unit and F.N.Ç.T. who is a cardiologist. It was applied for each measurement, and the probe was removed after the measurements. Measurements were conducted in accordance with the European Society of Cardiology guidelines.^3^ The TAPSE value shown in Figure 2A was calculated using transthoracic echocardiography (TTE) from the lateral annulus of the tricuspid valve in the apical four chamber view, using the MM mode, at the time of T_0_ and T_2_ (S-4 transducer with Philips Affiniti 30 System-Philips Healthcare, Andover, MA, USA).

RVEDA/LVEDA is shown in Figure 2B, and RV end diastolic volume (RVEDV) and LV end diastolic volume (LVEDV) are shown in Figure 2C, in mid esophageal four chamber image. The LV outflow tract (LVOT) diameter and area were measured and calculated on the mid-esophageal aortic valve long-axis image, as shown in Figure 2D. In Figure 2E, the LVOT velocity time integral (VTI) was measured with anteflexion in the deep transgastric (TG) axis using pulsed Wave Doppler. Stroke volume (SV) was calculated using the LVOT VTI and LVOT areas. CO was calculated using SV and heart rate (HR), as CO=(HRxSV).^4, 5^ LVESEI was calculated in the TG mid-papillary short axis image shown in Figure 2F provided with anteflexion in the TG axis.^6, 7^

During each measurement, mechanical ventilation parameters were recorded, vital signs were recorded, arterial blood gas analysis was performed, and Cstat and driving pressure (dP) values were measured. Using the PaO_2_:FiO_2_ to Horowitz ratio, DO_2_ values, COx(Hb×1.34×SaO_2_+PaO_2_×0.003) were recorded.

### Statistical Analysis

Statistical analyses were performed using the statistical software packages R (R Core Team, 2020) and Jamovi. Conformity of data to the normal distribution was evaluated using the Shapiro-Wilk test and Q-Q marking. Descriptive statistics are expressed as mean (standard deviation) for normally distributed numerical variables, median (interquartile range) for non-normally distributed numerical variables, and frequency (percent) for categorical variables. Numerical variables of two independent subgroups were compared using the Mann-Whitney U test for numerical data and the chi-square test for categorical data, with the Fisher’s exact test used as appropriate. The numerical variables of the two dependent groups were compared using the paired t-test, and the variables that did not fit the normal distribution were compared using the Wilcoxon test. Comparison of three dependent groups was made with repeated measures analysis of variance test, and the η^2^ G value was used as the effect size.^8^ Paired comparisons were made with Student’s t-test, and *P* values were corrected by Holm’s method. A *P *value of < 0.05 was considered statistically significant. Pearson’s correlation coefficients were calculated to determine the direction and strength of the relationship between the normally distributed numerical variables. A Kaplan-Meier analysis was used to evaluate the survival difference between two independent subgroups, and the log-rank test was used for comparisons between the two groups.

## Results

### Patient Population

Of the 61 patients with C-ARDS, 29 were excluded based on the exclusion criteria. Two patients were excluded because TEE deteriorated after T_0_ measurement and the measurements could not be continued. Thirty patients were included in the study, Figure 1. This study included 18 female (60%) and 12 male (40%) patients. The mean age was 65.5±10.9 years. Ninety percent of the patients were PCR positive and 100% had CT findings. According to the Berlin ARDS severity classification, 53.3% (n = 16) were moderate, 46.7% (n = 14) were severe. Intensive Care Unit (ICU) admission scores sequential organ failure assessment: 9.87±2.45, acute physiology and chronic health evaluation II: 28.5±7.12, charlson comorbidity index: 4.17±2.17, pneumonia severity index score: 88.9±27.3 are shown in Table 1. The comorbidities of patients on ICU admission are shown in Table 1; analysis of the parameters evaluated at T_0_-T_2_ and analysis of the cardiopulmonary parameters evaluated at T_0_-T_1_-T_2_ is shown in Table 2.

**Figure 3:**
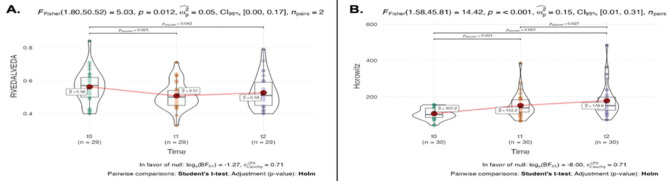
Change of RVEDA/LVEDA and Horowitz ratio with prone position

When the relationship between T_0_, T_1_, and T_2_ times of RVEDA/LVEDA was evaluated, it was determined that the decrease in values was significant (*P*=0.012). While the decrease in the 1^st^ hour after PP compared to pre-PP (*P*=0.025) and the decrease after the 24^th^ hour after PP (*P*=0.042) were significant, the difference between the 1^st^ and 24^th^ hours was not significant. A graph showing the mean values for T_0_: 0.56, T_1_: 0.51, T_2_: 0.53 (n = 29) is shown. The mean Horowitz value pre-PP was 107.2 and post-PP was 178.6 (*P *< 0.001). The increase occurred in all stages, with an average increase of 45 (units) from T_0_ to T_1_ and 26.4 (units) from T_1_ to T_2_. There was a significant increase in all the measurements (T_0_-_1_ *P*=0.001, T_0_-_2_ *P*=0.001, T_1_-_2_ *P*=0.027) (Figure 3).

**Figure 4:**
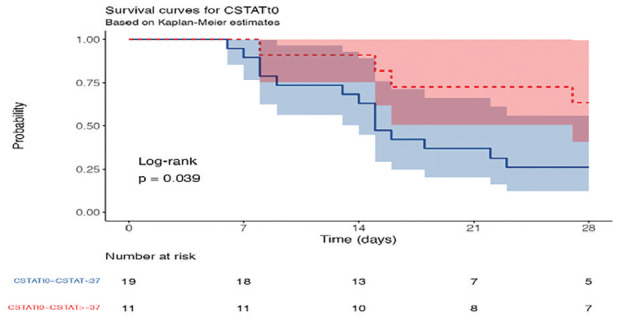
Survival curves for static compliance

A statistically significant correlation was found between the Cstat value and 28-day survival. According to the Kaplan-Meier calculation, the cut-off Cstat value was found to be 37, and the 28-day survival was lower in patients, with a value <37. At the end of 28 days, 5 of 19 patients with Cstat at T_0_ <37, and 7 of 11 patients with Cstat ≥37, survived. Each Cstat value from T_0_, T_1_, and T_2_ was found to be significant in terms of survival. It was determined that an increase of 1 unit according to Cstat at T_0_ value increases the probability of 28-day survival with an HR of 0.91 (0.86-0.98, *P*=0.007), (T_0_ t=2.913 *P*=0.007, T_1_ t=2.796 *P*=0.009, T_2_ t=3.267 *P*=0.003) (Figure 4).

**Figure 5:**
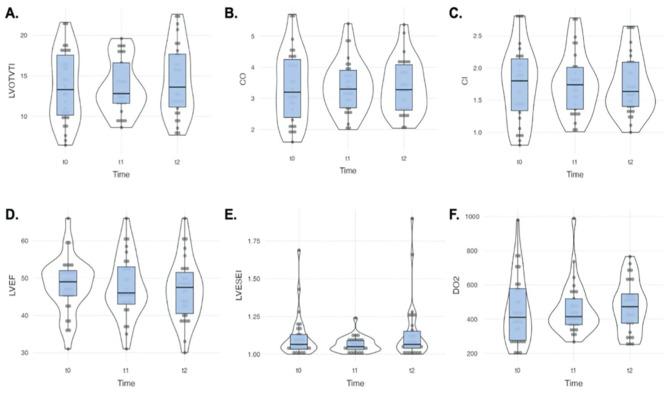
Change of LVOT-VTI, CO, CI, LVEF, LVESEI, DO_2_ with prone position CO, cardiac output; CI, cardiac index; LVEF, left ventricular efection fraction; LVESEI, left ventricular eccentricity index

Changes in the CO, confidence interval (CI), ejection fraction, LVOT VTI, LVESEI, DO_2_ values were not significant (Figure 5). There was no significant difference between patients who survived and those who did not in terms of the total number of PP hours applied over 28 days. Although lower T_1_ Horowitz and delta Horowitz between T_0_-T_1_ and T_0_-T_2_ were observed in the mortality group, these values were not significant.

## Discussion

In studies evaluating the heart-lung interaction in C-ARDS, the use of echocardiography has been recommended, with an emphasis on the importance of evaluating RV dysfunction (RVD) to reduce mortality.^9, 10, 11^ For the definition of RVD, among the parameters specified by the PRICES study published by the European Society of Intensive Care Medicine, the values of RVEDA/LVEDA and TAPSE were preferred.^12^ The recommended value of 0.6 (<0.6 normal, 0.6-1 dilated, >1 severe) is used as the RVEDA/LVEDA cut-off value for RVD definition.^13^ In the case series, which included nine C-ARDS patients with a Horowitz mean of 77, evaluations were conducted using TEE and three-dimensional (3D) before PP, at the first hour after PP, and in the supine position (PP+16 h). The RVEDA/LVEDA ratio did not increase; the LVESEI improved with PP; and the LVOT VTI decreased. The CI remained in balance with the increase in HR secondary to a decrease in LVOT VTI. RVEDV and LVEDV were observed to decrease significantly with the use of 3D.^14^

There was a small increase in LVOT VTI due to the effect of PP, which was not statistically significant. Reflecting the RVD recovery, RVEDA/LVEDA decreased, and TAPSE increased significantly. However, no increase in the CO was observed. This might be the result of LV worsening, concomitant with an improvement in the RVD. Chotalia et al.^15^ also showed that there are different cardiovascular sub-phenotypes in COVID-19 pneumonitis and that the PP response is different in sub-phenotypes. The significant increase in Horowitz was not sufficient to provide a significant change in the DO_2_ value. This showed that PP would not be sufficient to increase tissue oxygen supply, only by oxygenation, which could be possible with the combination of positive cardiac and pulmonary effects. Despite the severe C-ARDS, no significantly advanced RVD was observed. The median value of acute cor pulmonale (ACP) risk score was 3, but only 12 patients had a baseline RVEDA/LVEDA ≥0.6. Unless RVD causes LV dysfunction, its curative effect may not be sufficient to increase the DO_2_. The curative effect of PP on LV is observed when LV worsens secondary to RVD.

In a study evaluating the pulmonary circulation effects of inhaled nitric oxide (iNO) therapy in 12 C-ARDS patients with TTE, concomitant RV dilatation and dysfunction were demonstrated in only one-third of patients, despite baseline Horowitz values <150.^16^ The baseline Horowitz mean of our sample group of 30 was 107.2, and there were 12 patients with RVEDA/LVEDA ≥0.6. The improvement in RVD and oxygenation with PP shown in this study contrasts with findings from another RVD study, which did not show improvement with iNO treatment. RVD cannot be estimated using the Horowitz value, and the importance of echocardiography in diagnosis is clear. In improving oxygenation in C-ARDS patients, improvement in V/Q may contribute more than pulmonary vasodilatation. In another study in which sildenafil was used in the treatment of patients with C-ARDS, no significant improvement was found in oxygenation.^17^ The etiology of hypoxemia in C-ARDS varies and does not always cause increased pulmonary vascular resistance. Sometimes, pulmonary vasodilation is also a cause of hypoxemia.^18^ In a study stating that there is a relationship between an increase in CI and hypoxemia, a pulmonary artery catheter was used in the analysis, and increased shunt flow was stated as the cause of hypoxemia.^19^

In the study by Vieillard-Baron et al.^1^ with 42 ARDS patients, Vieillard-Baron et al.^1^ divided the patients into two groups, ACP and non-ACP, and evaluated them with TEE twice, before and after PP (PP+18 hours). In the ACP group, RVEDA/LVEDA improvement, CI increase, and LVESEI improvement were significant. Working on homogeneity with Horowitz <100 has proved advantageous. Had the patient group been divided into multiple groups according to the RVEDA/LVEDA value in our study, a better relation could have been observed by evaluating during PP, just before returning to the supine position, in addition to our assessments.

An analysis of the relationship between C-ARDS and RVD in 90 patients showed that longitudinal contraction of the RV was preserved, but there was radial damage.^20^ Similarly, the increase in TAPSE values with PP was significant, but the mean value of the baseline was 19.2±3.53, which was already preserved. Not measuring tricuspid regurgitation, systolic pulmonary artery pressure, or TAPSE’s inability to evaluate radial damage may have affected the result of TAPSE not being associated with survival.

RV free wall strain was evaluated in a study of 32 C-ARDS patients, and abnormal strain was observed in them. The compliance, and mechanical ventilation parameters were better in patients with low strain values. They concluded that RVD in C-ARDS develops from cardiac damage or vascular thrombosis rather than from pulmonary causes.^21^ They found a significant correlation between RVD and mortality in a cohort study using TEE, and examined the longitudinal shortening fraction as a prognostic factor in C-ARDS.^22^ Temperikidis et al.^23^ analyzed nine C-ARDS patients before PP, 18 hours after PP, and 1 hour after returning to supine. Abnormal onset strain values are a poor prognostic indicator.

In a multicenter cohort study by Vandenbunder et al.^24^ that included 1^st^ day and 14^th^ day values and was conducted with 372 patients, no 28-day survival relationship was observed, although there was a significant decrease in the value on the 14^th^ day. In a study examining the effect of PP on Horowitz and Cstat in C-ARDS and non-C-ARDS patients, the effects of the first PP were effective in predicting the prognosis.^25^ In the other study, they concluded that low Cstat and high D-dimer levels were associated with mortality.^26^ Here, a significant correlation between Cstat in the first 48 h after orotracheal intubation and 28-day survival in C-ARDS was observed.

Fossali et al.^27^ used electrical impedance tomography during PP, in a survival analysis of increased oxygenation in 21 patients with moderate/severe C-ARDS. They showed increased oxygenation, lung area gain in the dorsal areas, and a decrease in the dead space shunt ratio in the ventral areas, but found no significant correlation between increased lung area and either disease severity or improvement in oxygenation.^27^ Despite the increase in oxygenation, we did not see a significant increase in DO_2_ in our study. Among the pulmonary parameters affected by PP, dP decreased, and Cstat and Cdyn increased. Cstat, a cardiopulmonary parameter that we found to be significantly related to survival, draws attention to the importance of a compatible lung. The importance of the compatible lung can be explained both by the protective effect on the lung by preventing pressure and volume damage, and by its protective effect on the heart through the heart-lung interaction.

### Study Limitations

Limitations of this study; two-dimensional was used in volume evaluation, the number of our patient group and it was single-centered. The effect of the PP on the RV in C-ARDS is better evaluated in comparison to patients without PP. In this study, where the relationship between RVD and survival was examined, we believe that the presence of additional factors affecting the results. Indeed, in most patients, secondary infections and the development of septic shock were causes of mortality.

## Conclusion

The cardiopulmonary pathophysiology and outcomes of C-ARDS are variable, and this variability requires monitoring throughout the diagnosis and treatment process. PP can improve RV recovery and oxygenation; however, it does not always lead to increased survival. The use of echocardiography is important in evaluating the mechanism of cardiopulmonary injury and treatment process in C-ARDS patients with frequent RVD and high mortality, and its use is becoming common. The fact that the increase in Cstat has been shown to be associated with 28-day survival suggests that a better-functioning lung will interact more effectively with the heart and improve survival. Randomized, multicenter studies are needed on this subject. It could be said that holistic recovery and individualized treatment strategies should be targeted instead of improvement in a single parameter.

## Ethics

**Ethics Committee Approval:** This study was approved by the University of Health Sciences Türkiye, Bakırköy Dr. Sadi Konuk Training and Research Hospital, Clinical Research Local Ethics Committee with the decision number 2022-03-03, dated 07.02.2022.

**Informed Consent:** Informed consent was obtained.

## Figures and Tables

**Table 1. Patient’s Characteristics and Comorbidities on ICU Admission:** 

-	**Total n = 30**
	**(SD)**
Age (years)	64.5 (10.9)
PBW (kg)	57.6 (10.5)
BMI (kg m^2-1^)	33.4 (9.03)
BSA (m^2^)	1.93 (0.144)
SOFA score	9.87 (2.45)
APACHE II score	28.5 (7.12)
CCI score	4.17 (2.17)
PSI score	88.9 (27.3)
ACP risk score	3 [1]
Timing of OTI (days from diagnosis)	11.5 (9.45)
Duration of IMV (days)	15.1 (8.55)
LOS in ICU (days)	18.5 (9.31)
LOS in hospital (days)	26.1 (14.2)
Survival (days)	20.8 (9.67)
Total PP hours	135 (83.3)
-	** n (%)**
Severe ARDS	14 (46.7)
Diabetes mellitus	11 (36.7)
Hypertension	15 (50)
Coronary artery disease	6 (20)
Lymphoma	4 (13.3)
Asthma	3 (10)
Solid tumor	4 (13.3)
Chronic kidney disease	2 (6.7)
Leukemia	6 (20.0)
No smoke	17 (56.7)
Mortality-28 day	18 (60)

Values are presented as mean ± SD or incidence (%).

PBW, predicted body weight; BMI, body mass index; BSA, body surface area; SOFA, sequential organ failure assessment; APACHE II, acute physiology and chronic health evaluation II; CCI, Charlson comorbidity index; PSI, pneumonia severity index; ACP, acute care pathway; OTI, orotracheal intubation; IMV, invasive mechanical ventilation; LOS, length of stay; ICU, intensive care unit; PP, prone position; ARDS, acute respiratory distress syndrome

**Table 2. Analysis of the Cardiopulmonary Parameters Evaluated in T0-T2 and T0-T1-T2:** 

-	**T_0_ (pre-PP)**	**T_2 _(T_0_+24 h)**	-	-	-	-
	**(SD)**	** (SD)**	**t**	**P**	**d**	-
**TAPSE (mm) (n = 25)**	19.2 (3.53)	20.4 (2.31)	-2.13	**0.044**	0.425	-
-	**Median (IQR)**	**Median (IQR)**	**W**	***P***	**rbc**	-
**Troponin (ng L^-1^)**	27.8 [43.6]	33.3 [42.9]	132	0.066	0.393	-
**pro-BNP (ng L^-1^) (n = 29)**	1122 [2222]	1074 [2895]	199	0.701	-0.085	-
**CK (U L^-1^)**	118 [228]	80 [84.8]	316	**0.035**	0.451	-
-	T_0 _(pre-PP)	T_1 _(T_0_+1 h)	T_2 _(T_0_+24 h)	-	-	-
-	** (SD)**	** (SD)**	** (SD)**	**F**	***P***	**η**^2^**G**
**RVEDV (mL) (n = 27)**	42.3 (16.5)	31.8 (11)	33.4 (13.6)	15.6	**<0.0001**	0.091
**LVEDV (mL) (n = 29)**	96.0 (33.2)	84.4 (24.8)	88.6 (24.9)	4.07	**0.022**	0.033
**LVESV (mL)**	50 (19.7)	43.4 (12.3)	46.8 (15.1)	3.8	**0.028**	0.036
**SAP (mmHg)**	133 (26.6)	124 (17.0)	135 (23.8)	2.27	0.112	0.041
**DAP (mmHg)**	65.3 (13.8)	62.5 (11.0)	65.3 (9.46)	0.969	0.386	0.014
**MAP (mmHg)**	88.7 (16.7)	84.4 (14.3)	90.4 (14.6)	1.58	0.215	0.028
**Heart rate (beat mn)**	89.6 (26.4)	88.7 (20.8)	87.6 (22.1)	0.147	0.864	0.001
**Norepinephrine (µg kg^-1 ^min)**	0.100 (0.061)	0.108 (0.074)	0.156 (0.183)	0.066	0.936	0.002
**Balance (mL)**	286 [662]	367 [969]	568 [1138]	12.2	**0.002**	-
**SPO_2 _(%)**	93.4 (3.26)	94.7 (2.48)	94.1 (2.05)	1.41	0.257	0.036
**FiO_2 _(%)**	0.782 (0.114)	0.689 (0.141)	0.617 (0.162)	24.5	**<0.001**	0.193
**PaO_2_ (mmHg)**	81.9 (19.8)	98.6 (28.6)	99.3 (26.5)	5.1	**0.009**	0.095
**PaCO_2_ (mmHg)**	50.6 (13.6)	50.8 (10.6)	56.6 (21.8)	1.46	0.241	0.031
**MV (L mn)**	7.10 (1.34)	7.31 (1.18)	6.99 (1.32)	0.891	0.416	0.011
**TV (mL)**	468 (82.4)	470 (62.8)	454 (68.8)	1.14	0.326	0.011
**Peep (cmH_2_O)**	9.00 [2.00]	9.50 [2.00]	10 [2.00]	1.08	0.582	-
**dP (cmH_2_O) (n = 29)**	17.0 (3.89)	17.3 (3.50)	15.7 (2.93)	4.39	**0.017**	0.030
**Cdyn (mL cmH_2_O)**	29.4 (9.13)	28.0 (6.21)	30.2 (9.47)	4.19	**0.020**	0.010
**Cstat (mL cmH_2_O)**	33.0 (9.85)	30.7 (8.92)	34.0 (10.1)	7.72	**0.001**	0.020
**pH**	7.36 (0.106)	7.35 (0.101)	7.34 (0.120)	0.325	0.724	0.005
**BE (mmoL L^-1^)**	2.36 (4.52)	1.76 (4.42)	3.47 (4.34)	4.06	**0.022**	0.026
**Lactate (mmoL L^-1^)**	1.75 [0.950]	1.70 [0.650]	1.60 [0.875]	2.75	0.252	-
**SO_2_ %**	94.2 [4.77]	96.4 [4.25]	96.4 [2.25]	14.6	**<0.001**	-
**Bicarbonate (mmoL L^-1^)**	26.1 (4.27)	25.4 (4.19)	26.8 (4.34)	2.92	0.062	0.020

Values are presented as mean ± SD or median.

t, Paired samples t-test; d, Cohen’s d effect size; P, Probability, *P* < 0.05

TAPSE, tricuspid annular plane systolic excursion; IQR, interquartile range, rbc, rank biserial correlation; BNP, B-type natriuretic peptide; W, Wilcoxon; CK, creatine kinase; PP, prone position; F, F test; η^2^G, Effect size; RVEDV, right ventricular end diastolic volume; LVEDV, left ventricular end diastolic volume; LVESV, left ventricular end systolic volume; SAP, systolic arterial pressure; DAP, diastolic arterial pressure; MAP, mean arterial pressure; MV, minute volume; TV, tidal volume; dP, driving pressure; Cdyn, compliance of dynamic; Cstat, compliance of static; BE, base excess.
